# The Benefits of a Rehabilitation Program Following Medial Patellofemoral Ligament Reconstruction

**DOI:** 10.3390/life14111355

**Published:** 2024-10-23

**Authors:** Claudia-Camelia Burcea, Maria-Daniela-Antonia Oancea, Diana-Lidia Tache-Codreanu, Luminița Georgescu, Ioana-Cristina Neagoe, Corina Sporea

**Affiliations:** 1 Faculty of Midwifery and Nursing, University of Medicine and Pharmacy “Carol Davila”, 37 Dionisie Lupu Street, 020021 Bucharest, Romania; claudia_burcea@yahoo.com (C.-C.B.); corina.sporea@gmail.com (C.S.); 2 Oancea Maria-Daniela-Antonia Physiotherapy Activities, 1 Brebu Street, 023671 Bucharest, Romania; antoniaoancea.d@gmail.com; 3 Medical Rehabilitation Department, Colentina Clinical Hospital, 19–21 Stefan cel Mare Street, 020125 Bucharest, Romania; 4 Faculty of Science, Physical Education and Informatics, University Center of Pitești, National University of Science and Technology Politehnica Bucharest, 7 Aleea Şcolii Normale, 110254 Pitești, Romania; lgeorgescu0507@upb.ro (L.G.); ioana.neagoe@upb.ro (I.-C.N.); 5 National Teaching Center for Children’s Neurorehabilitation “Dr. Nicolae Robanescu”, 44 Dumitru Minca Street, 041408 Bucharest, Romania

**Keywords:** medial patellofemoral ligament reconstruction, medial patellofemoral ligament, physiotherapy, rehabilitation, pain management, knee function

## Abstract

The medial patellofemoral ligament (MPFL) is critical for patellar stability. This study investigates the efficacy of a one-year physical therapy rehabilitation program following MPFL reconstruction using Synthetic Graft (SG) and Quadriceps Tendon Autograft (QTA). Thirty-five patients aged 18–38 underwent MPFL reconstruction (20 SG, 15 QTA). They participated in a structured rehabilitation program to improve their range of motion (ROM), muscle strength, pain management, and overall quality of life (QoL). The program included physiotherapy and MLS laser, Game Ready Therapy, EMS, TENS, TECAR, and lymphatic drainage. Before and after the program, assessments included knee flexion and extension using goniometry, muscle strength via the Medical Research Council (MRC) scale, knee circumference, pain intensity on the Visual Analogue Scale (VAS), and QoL with the EQ-5D instrument. Significant improvements were observed in knee flexion (37.57° vs. 114.71°, *p* < 0.001), muscle strength (MRC scale 1–4 points vs. 4–5 points, *p* < 0.001), and pain reduction (VAS 6.66 vs. 0.46, *p* < 0.001). The functional coefficient of mobility and QoL scores also markedly increased. Patients with QTA improved some parameters better than those with SG. These findings support the effectiveness of a comprehensive rehabilitation program in enhancing knee functionality, reducing pain, and improving QoL post-MPFL reconstruction. Personalized rehabilitation protocols are recommended to optimize recovery outcomes.

## 1. Introduction

The medial patellofemoral ligament (MPFL) is a crucial structure in the knee joint, playing a primary role in stabilizing the patella (kneecap) and preventing lateral dislocations [1,2,3]. Anatomically, it originates from the medial femoral epicondyle, adductor tubercle, and gastrocnemius tubercle, and inserts on the superomedial margin of the patella [4]. The anterior fibers of the MPFL interweave with the deep fibers of the vastus medialis muscle [5,6]. Lateral patellar dislocation is a frequent and severe knee injury that leads to diminished knee functionality, lower quality of life, and ongoing activity restrictions [7]. Acute lateral patellar dislocations are often associated with MPFL ruptures [4]. The most common mechanism of injury involves an indirect force, typically an external rotation of the tibia relative to the femur with the foot fixed on the ground [8]. These dislocations are most prevalent among young individuals and adolescents, often resulting from sports-related trauma [9,10,11].

MPFL reconstruction for patellar instability is linked to outstanding clinical results and a high rate of return to play, with a low occurrence of recurrent dislocation or instability [12]. Postoperative rehabilitation is typically conducted after surgery, and having safe and effective programs is crucial for achieving successful clinical results and enabling a return to daily and athletic activities. These programs focus on enhancing the range of motion and muscle strength, reducing pain, and decreasing edema, evidenced by the normalization of the supra-, peri-, and suprapatellar diameters, ultimately improving quality of life [13]. From a medical perspective, QoL assessments reflect improvements in health status and the effectiveness of therapeutic–rehabilitative interventions [14].

Surgical reconstruction of the MPFL is generally considered after at least two patellar dislocations [15,16]. Additionally, surgery is the preferred treatment for primary patellar dislocations associated with large displaced osteochondral fractures or complete avulsions of the vastus medialis obliquus muscle [17,18]. The choice and method of graft fixation are paramount for achieving optimal knee function [15,16,19].

Incomplete or inadequate recovery following MPFL reconstruction can lead to long-term complications and an increased risk of further knee pathologies, significantly impacting knee stability and function within the lower extremity kinetic chain. Recurrent pain episodes can also diminish a patient’s independence in daily activities and, consequently, their quality of life.

The postoperative rehabilitation program aims at regaining ROM, strength, and neuromuscular coordination, achieving painlessness and stability of the knee after MPFL reconstruction.

## 2. Materials and Methods

The primary objective of this retrospective study was to demonstrate the benefits of a physical therapy rehabilitation program following MPFL reconstruction. The program’s results were evaluated in terms of pain reduction, restoration of range of motion and joint mobility, muscle strengthening around the knee joint, improvement of proprioception and balance, and facilitation of return-to-normal activities.

A group of 35 patients who underwent MPFL reconstruction, 20 with Synthetic Graft (SG) and 15 with Quadriceps Tendon Autograft (QTA), followed a 1-year rehabilitation program for knee functional rehabilitation. The inclusion criteria were an age between 18 and 38 years, and participation in the rehabilitation program after MPFL reconstruction. The exclusion criteria were patients under the age of 18 or over the age of 38, and patients with polytrauma who had to undergo different medical rehabilitation programs for multiple injuries simultaneously.

The rehabilitation program consisted of physical therapy [20] and additionally included the following:

- Laser Multiwave Locked System (MLS) for pain relief, reduction in swelling and edema, and improvement in vascular trophycity [21,22].
- Game Ready Therapy combining cold therapy and dynamic compression for pain relief, reduction in swelling and edema, and facilitation of venous and lymphatic return circulation [23,24].
- Electrical Muscle Stimulation (EMS) to increase muscle strength [25].
- Transcutaneous Electrical Nerve Stimulation (TENS) to relieve pain and reduce muscle contractions [26].
- Capacitive Resistive Energy Transfer (TECAR) for pain relief, reduction in swelling and edema, improvement in blood circulation, and muscle relaxation [27,28].
- Lymphatic drainage for reduction in swelling and the facilitation of venous and lymphatic return circulation.

The rehabilitation program had four stages, as presented in Table 1.

The main objective of this study was to evaluate the effectiveness of the interventions used in physiotherapy programs for regaining the range of joint mobility, strength, and neuromuscular coordination, achieving painlessness and stability of the knee after MPFL reconstruction. The following parameters were assessed at two different moments (before starting and after finishing the rehabilitation program):

- Knee flexion and extension range of motion using goniometry.
- Functional coefficient of mobility (CFM): Different mobility angles are assigned a utility coefficient based on their usefulness and functional attitudes, with the highest coefficients allocated to the angles most favorable to functionality. The maximum value, 100%, represents the ideal and highest coefficient for a joint [29].
- Muscle strength using the Medical Research Council (MRC) Scale for Muscle Strength [30].
- Knee perimeters: Peripatellar (as an edema marker) and subpatellar and suprapatellar (as muscle tone markers).
- Pain on the Visual Analogue Scale (VAS) [31].
- Quality of Life following MPFL reconstruction, assessed with EQ-5D, an instrument that assesses five dimensions: mobility, self-care, usual activities, pain/discomfort, and anxiety/depression. Each dimension has five levels: no problems, mild problems, moderate problems, severe problems, and extreme problems. The score ranges from 0 to 100 points, with 100 representing no problems and 0 representing extreme problems [32,33].

Statistical calculations were performed using Microsoft Excel (Microsoft Corporation, Redmond, WA, USA) for data preparation and SPSS 24.0 version for Windows (IBM Inc., Chicago, IL, USA) for the analysis. The *t*-test was applied after checking data normality distribution with the Shapiro–Wilk test. The Paired-Samples *t*-test was used to assess differences between groups and effect size. A *p*-value less than 0.05 was considered statistically significant, and the confidence level was 95%, with related intervals corresponding to the calculated averages [34,35,36].

## 3. Results

The study group comprised 35 patients who underwent MPFL reconstruction (43% with QTA and 57% with SG) and followed a physical rehabilitation program. Among the participants, 51% were men and 49% were women. Additionally, 49% had the right side affected, while 51% had the left side affected. The ages ranged from 18 to 38, with an average of 25.74 years.

**Figure 1:**
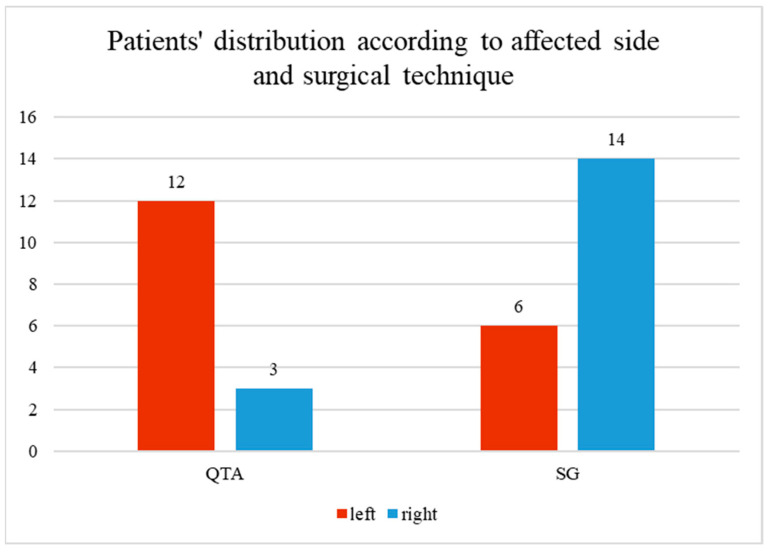
Patients’ distribution according to surgical technique and affected side.

Figure 1 illustrates the distribution of patients based on the affected side (left or right knee) and the surgical technique employed. Two surgical techniques were used: Quadriceps Tendon Autograft (QTA) and synthetic graft (SG). For the QTA group, 12 patients had surgery on the left knee, and 3 on the right knee. In the SG group, 6 patients had surgery on the left knee, and 14 on the right knee. The red bars represent the number of patients with the left knee affected, while the blue bars represent those with the right knee affected.

We monitored several key parameters to assess the effectiveness of the rehabilitation program following MPFL reconstruction. These parameters included knee flexion, extension deficit, functional coefficient of mobility, muscle strength, knee perimeters (subpatellar, peripatellar, suprapatellar), pain, and the five subdomains of quality of life: mobility, self-care, usual activities, pain, and anxiety. Since normality tests indicated that the data did not follow a normal distribution, we present the results as median values, along with the interquartile range (IQR) and minimum–maximum intervals, providing a clear depiction of the central tendency and data spread for each parameter. Table 2 presents the median, interquartile range (IQR), and minimum–maximum intervals for each monitored parameter, including knee functionality metrics and quality-of-life subdomains, in patients who followed the rehabilitation program.

### 3.1. Knee Flexion Before and After the Rehabilitation Program

**Figure 2:**
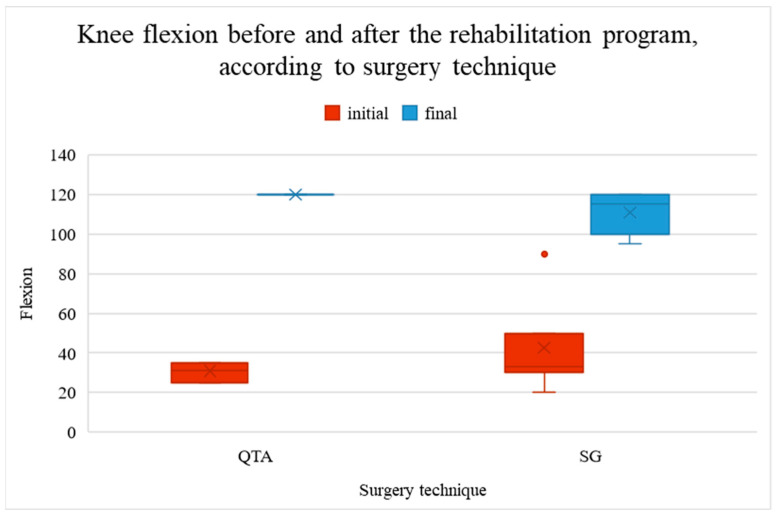
Knee flexion before and after the rehabilitation program.

Knee flexion was significantly lower (worse) before the rehabilitation program (mean value 37.57° vs. 114.71°, *p* < 0.001). Patients with QTA showed better improvement (89.13°) than those with SG (68.15°), as shown in Figure 2.

Figure 2 represents the knee flexion measurements before and after the rehabilitation program, categorized by surgical technique. The red boxes display the initial knee flexion (pre-rehabilitation), while the blue boxes show the final knee flexion (post-rehabilitation). Two surgical techniques are compared: Quadriceps Tendon Autograft (QTA) and synthetic graft (SG). For both techniques, there is a significant improvement in knee flexion following rehabilitation. The median and interquartile ranges (IQR) are depicted, with outliers represented as individual points. The ‘x’ markers inside the boxes represent the mean values for each group. Knee flexion is measured in degrees, with higher values indicating greater knee mobility.

### 3.2. Knee Extension Deficit Before and After the Rehabilitation Program

**Figure 3:**
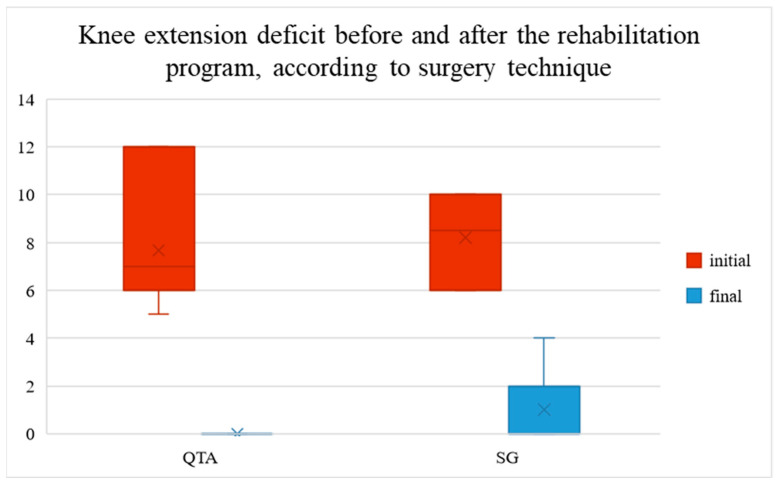
Knee extension deficit before and after the rehabilitation program.

The initial knee extension deficit ranged between 5 and 12° (5–12° in patients with QTA and 6–10° in patients with SG). It decreased to a mean value of 7.67° in patients with QTA and 7.20° in patients with SG, ranging between 0 and 4° after the rehabilitation program (0° in patients with QTA, 0–4° in patients with SG), as shown in Figure 3.

Figure 3 shows the knee extension deficit before and after the rehabilitation program, categorized by surgical technique. The red bars represent the initial knee extension deficit (pre-rehabilitation), while the blue bars display the final deficit (post-rehabilitation). Two surgical techniques are compared: Quadriceps Tendon Autograft (QTA) and synthetic graft (SG). The chart indicates a notable reduction in extension deficit after rehabilitation. The height of the bars reflects the median values, with the interquartile range (IQR) shown for variability. The ‘x’ marks within the bars indicate the mean values. Lower values indicate smaller deficits, suggesting better knee extension recovery.

### 3.3. Functional Coefficient of Mobility Before and After the Rehabilitation Program

**Figure 4:**
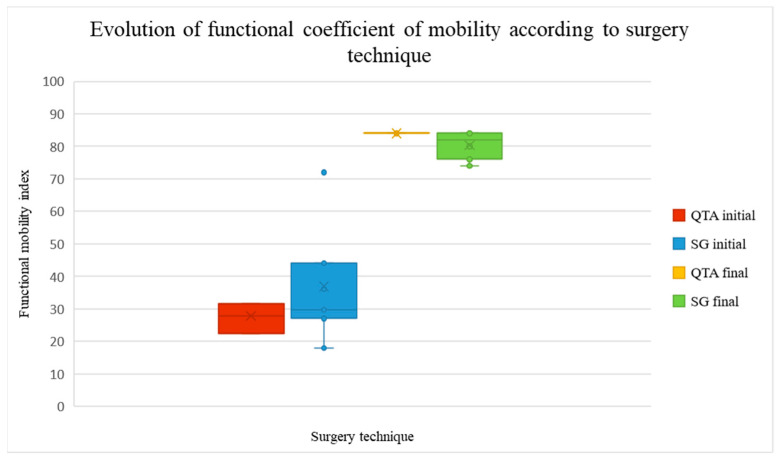
Evolution of knee functional coefficient of mobility.

The functional coefficient of mobility increased after the rehabilitation program from 18 to 72% (22.5–31.5% in patients with QTA and 18–72% in patients with SG) to 74–84% (84% in patients with QTA, 74–84% in patients with SG), as shown in Figure 4.

Figure 4 illustrates the evolution of the knee functional coefficient of mobility (CFM) before and after the rehabilitation program, categorized by surgical technique. The red and blue bars represent the initial CFM (pre-rehabilitation), while the yellow and green bars display the final CFM (post-rehabilitation). Two surgical techniques are compared: Quadriceps Tendon Autograft (QTA)—red and yellow—and synthetic graft (SG)—blue and green. The chart indicates a notable increase in CFM after rehabilitation, particularly for the QTA group. The height of the bars reflects the median values, with the interquartile range (IQR) illustrating data variability. The ‘x’ marks within the bars indicate the mean values. Higher CFM values suggest better mobility.

### 3.4. Muscle Strength Before and After the Rehabilitation Program

**Figure 5:**
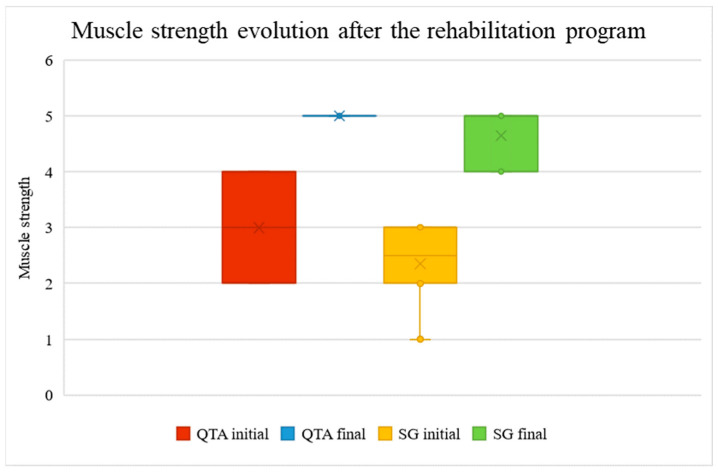
Muscle strength evolution after the rehabilitation program.

Patients experienced an increase in muscle strength after the rehabilitation program. The muscle strength was measured on the MRC scale (2–4 points before vs. 5 points after in patients with QTA, and 1–3 points before vs. 4–5 points after in patients with SG *p* < 0.001), as shown in Figure 5.

Figure 5 represents muscle strength measurements before and after the rehabilitation program, categorized by surgical technique. The red and yellow boxes display the initial muscle strength (pre-rehabilitation), while the blue and green boxes show the final muscle strength (post-rehabilitation). Two surgical techniques are compared: Quadriceps Tendon Autograft (QTA)—red and blue—and synthetic graft (SG)—yellow and green. For both techniques, there is a significant improvement in muscle strength following rehabilitation. The median values and interquartile ranges (IQR) are depicted, with outliers represented as individual points. The ‘x’ markers inside the boxes indicate the mean values for each group. Higher values reflect greater muscle strength.

### 3.5. Knee Circumference Before and After the Rehabilitation Program

**Figure 6:**
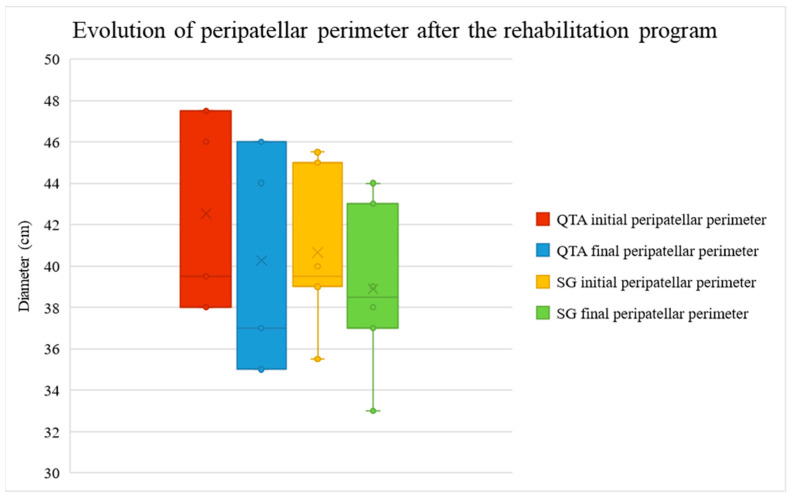
Peripatellar perimeter evolution after the rehabilitation treatment.

The peripatellar perimeter, assessed as an edema marker at knee level, decreased in QTA patients from the mean value of 42.53 cm (minimum 38, maximum 47.5 cm) to 40.27 cm (minimum 35, maximum 46 cm) and in SG patients from 40.65 (minimum 35.5, maximum 45.5 cm) cm to 38.90 cm (minimum 33, maximum 44 cm), as shown in Figure 6.

Figure 6 illustrates the peripatellar perimeter before and after the rehabilitation program, categorized by surgical technique. The red and yellow boxes display the initial peripatellar perimeter (pre-rehabilitation), while the blue and green boxes show the final peripatellar perimeter (post-rehabilitation). Two surgical techniques are compared: Quadriceps Tendon Autograft (QTA)—red and blue—and synthetic graft (SG)—yellow and green. For both techniques, there is significant edema reduction following rehabilitation. The median values and interquartile ranges (IQR) are depicted, with outliers represented as individual points. The ‘x’ markers inside the boxes indicate the mean values for each group. Lower values indicate greater edema reduction.

**Figure 7:**
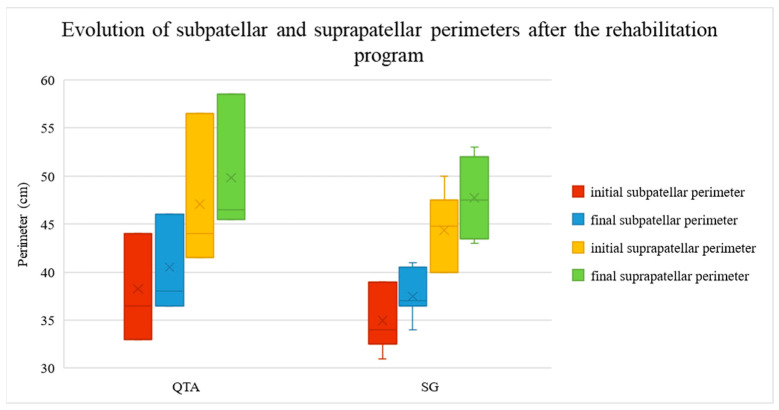
Sub- and suprapatellar perimeter evolution after the rehabilitation treatment.

Subpatellar and suprapatellar perimeter, assessed as muscle tonus marker at knee level, increased in both QTA and SG patients after the rehabilitation program. Initial subpatellar perimeter values ranged between 33 and 44 cm, with a mean value of 38.27 for QTA patients, and between 31 and 39 cm with a mean value of 34.98 cm for SG patients. The final subpatellar perimeter values ranged between 36.5 and 46 cm, with a mean value of 40.53 for QTA patients, and between 34 and 41 cm with a mean value of 37.5 cm for SG patients. Initial suprapatellar perimeter values ranged between 41.5 and 56.5 cm with a mean value of 47.07 cm for QTA patients and between 40 and 50 cm with a mean value of 44.38 cm for SG patients. The final suprapatellar perimeter values ranged between 45.5 and 58.5 cm with a mean value of 49.83 for QTA patients and between 43 and 53 cm with a mean value of 47.73 cm for SG patients. The evolution of subpatellar and suprapatellar perimeters is shown in Figure 7.

Figure 7 illustrates the subpatellar and suprapatellar perimeters before and after the rehabilitation program, categorized by surgical technique. The red and yellow boxes display the initial subpatellar and suprapatellar perimeters (pre-rehabilitation), while the blue and green boxes show the final subpatellar and suprapatellar perimeters (post-rehabilitation). Two surgical techniques are compared: Quadriceps Tendon Autograft (QTA)—left, and synthetic graft (SG)—right. For both techniques, there is significant improvement in muscle tone following rehabilitation. The median values and interquartile ranges (IQR) are depicted, with outliers represented as individual points. The ‘x’ markers inside the boxes indicate the mean values for each group. Higher values reflect greater muscle tone improvement.

### 3.6. Pain Intensity Before and After the Rehabilitation Program

Pain intensity was evaluated using the VAS and showed significant improvement (*p* < 0.001) after treatment pain, decreasing from 6.66 (6.80 in QTA patients and 6.55 in SG patients) to 0.46 (0 in QTA patients and 0.80 in SG patients).

### 3.7. Quality of Life Before and After the Rehabilitation Program

After completing the rehabilitation program, the patients’ QoL showed significant improvements in all five dimensions of the EQ-5D, as shown in Table 3.

**Figure 8:**
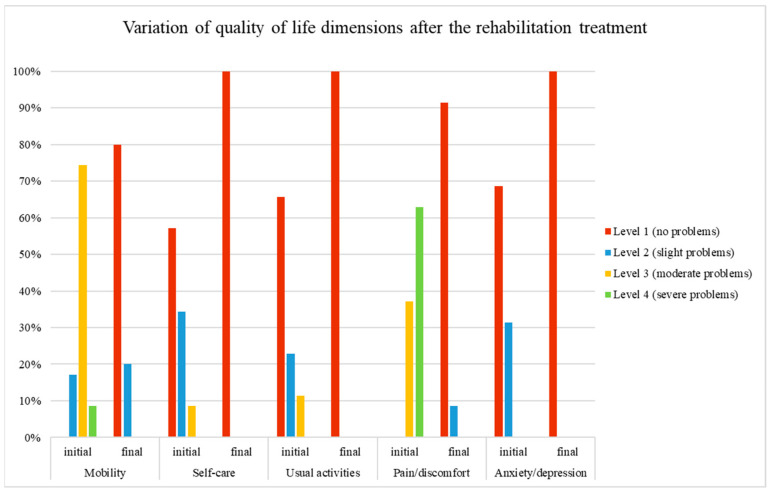
Quality of life dimensions before and after the rehabilitation program.

Figure 8 illustrates the variation in each of the five dimensions of quality of life (mobility, self-care, usual activities, pain/discomfort, and anxiety) before and after the rehabilitation program, categorized by level. The levels are represented by four values: 1 (red) = no problems, 2 (blue) = slight problems, 3 (yellow) = moderate problems, and 4 (green) = severe problems. Significant improvement is observed in all dimensions of quality-of-life following rehabilitation.

After the rehabilitation program, all patients reported no problems in self-care, usual activities, and anxiety/depression. Mobility and pain/discomfort also showed significant improvement. Before treatment, 83% of patients reported moderate to severe mobility problems, which decreased to only 20% reporting slight problems after treatment. Similarly, pain severity decreased from 63% experiencing severe problems and 37% having moderate problems before treatment to 9% reporting slight problems afterward.

Pearson Chi-square test for each domain was performed.

Pearson Chi-square χ^2^ (2, N = 35) = 13.846, *p* = 0.001 indicates a significant association between initial (pre-rehabilitation) and final (post-rehabilitation) mobility levels (Table 4). The Likelihood Ratio (LR = 12.703, *p* = 0.002) further supports this conclusion, reinforcing the significant association between the initial and final mobility levels. The Linear-by-Linear Association (LLA = 9.000, *p* = 0.003) suggests a significant linear trend, indicating that as initial mobility levels change, final mobility levels also tend to change in a consistent direction.

Pearson Chi-square χ^2^ (1, N = 35) = 1.939, *p* = 0.164 suggests that there is no statistically significant association between initial (pre-rehabilitation) and final (post-rehabilitation) pain levels (Table 5). This conclusion is reinforced by the continuity correction (*p* = 0.443). The Likelihood Ratio (LR test, *p* = 0.086) also suggests that there is no significant difference in the distribution of pain categories between the initial and final assessments. Fisher’s Exact Test further confirms this, with *p* > 0.05, indicating no significant association between the variables. Additionally, the Linear-by-Linear Association (LLA = 1.884, *p* = 0.170) shows no significant trend or association between initial and final pain levels.

For self-care, usual activities, and anxiety/depression, the Pearson Chi-square test indicated no association between the initial (pre-rehabilitation) and final (post-rehabilitation) values of these parameters.

## 4. Discussion

The findings of this study demonstrate that the rehabilitation program significantly improved knee function and muscle strength, reduced pain and edema, and enhanced quality of life in patients who underwent medial patellofemoral ligament (MPFL) reconstruction using either synthetic graft (SG) or quadriceps tendon autograft (QTA), consistent with the existing literature [37,38,39]. Although patients with QTA exhibited slightly better results in certain parameters, the improvements in knee flexion, extension, muscle strength, and functional mobility were significant and comparable between the two groups, as reported in other studies [40,41,42]. These findings align with previous research emphasizing the importance of structured and intensive rehabilitation programs following MPFL reconstruction [43,44].

The early establishment of a physiotherapy program after the reconstruction of the medial patellofemoral ligament (MPFL) and its phasing according to the type of graft used, the sequence of the graft integration processes, and the individual possibilities of healing and recovery of the patients of the study group demonstrated significant improvements in the amplitudes of joint movement, muscle strength and endurance, and joint stability, as well as the significant reduction in pain in the patients of the study group. Accelerated rehabilitation protocols, which involve little to no postoperative bracing and minimal weight-bearing restrictions, have demonstrated favorable outcomes when compared to more conservative approaches [45,46].

The appropriate combination of kinetic means, techniques, and methods with physiotherapy procedures led to the early recovery of the knee joint function and, at the same time, to the increase in the patient’s compliance with the treatment following.

The progression of muscle toning exercises facilitated the adequate integration of the soft tissue graft to the bone structures after the reconstruction of the medial patellofemoral ligament (MPFL), a fact demonstrated by the consistently good results obtained by the study group patients during the recovery program.

The proposed recovery program led to the uncomplicated reintegration of patients with medial patellofemoral ligament (MPFL) reconstruction into competitive or recreational sports activities, which improved the patients’ quality of life.

Appropriate knee joint functional recovery strategy after medial patellofemoral ligament reconstruction is as crucial as surgery in wholly and correctly managing recurrent patellar dislocation, considering its vital effect on knee joint biomechanics/functionality. The physical therapist’s decision to move from one stage of recovery to another must be based on the goals achieved by the patient in each stage, not only in time, taking into account the specifics of the patellofemoral joint.

### Limitations

The selection of patients exclusively from a rehabilitation department, where all participants followed a structured rehabilitation program after MPFL reconstruction, limited our ability to establish a control group of patients who did not undergo a rehabilitation protocol. Additionally, the absence of other relevant data, such as BMI, additional medical parameters, or a detailed medical history, may have influenced the conclusions of this study. To enhance the validity of future findings, a more comprehensive dataset will be collected. Moreover, prospective studies incorporating a control group would provide a stronger foundation for validating these results.

Regarding the Chi-square tests for mobility, although there is a significant association between initial and final mobility scores, suggesting that the rehabilitation program had a meaningful impact, four cells (66.7%) have an expected count less than 5. This indicates that the data may not fully meet the assumptions of the Chi-square test, as more than 20% of the expected frequencies fall below 5, which can impact the reliability of the test results.

Similarly, the Chi-square tests for pain/discomfort indicate that two cells (50.0%) have expected counts below 5. This suggests that half of the expected counts in the contingency table are insufficient, which could potentially affect the validity of the results. Future studies should consider using larger sample sizes or alternative statistical methods to address these limitations.

## 5. Conclusions

The findings of our study confirm that a structured physiotherapy rehabilitation program significantly improves knee joint mobility, muscle strength, pain relief, and quality of life in patients who have undergone MPFL reconstruction. These positive outcomes emphasize the critical role of individualized rehabilitation programs that incorporate advanced physical therapy techniques. Future research should focus on refining these protocols and investigating long-term outcomes to further optimize patient care and recovery.

## Figures and Tables

**Table 1:** The stages of the rehabilitation program and the goals of each stage.

Stage	Goals
1st Stage (Weeks 0–6)	Manage pain and inflammation; prevent venous return disorders; reduce soft tissue swelling; improve knee joint mobility; prevent and correct improper positions.
2nd Stage (Weeks 6–12)	Improve knee joint mobility (complete recovery of active and passive flexion ROM, extension = 0°); recover normal walking; increase muscle strength; recover passive and active stability of the knee in both bipodal and unipodal support; recover muscle volume (circumference difference between the healthy and affected limb < 1 cm).
3rd Stage (Months 3–6)	Improve knee joint mobility; increase muscle strength; complete recovery of muscle volume (circumference difference between the healthy and affected limb = 0 cm); develop sports skills.
4th Stage (Months 6–12)	Train lower limb muscles for reintegration into sports activity.

**Table 2:** Summary of monitored parameters in patients following MPFL reconstruction.

Parameter			Median	IQR	Range
Knee flexion (°)		Initial	33	10	20–90
		Final	120	10	95–120
Knee extension deficit (°)		Initial	8	4	5–12
		Final	0	0	0–4
Functional coefficient of mobility		Initial	29.7	9	18–72
		Final	84	4	74–84
Muscle strength		Initial	3	1	1–4
		Final	5	0	4–5
Perimeter (cm)	Peripatellar	Initial	39.5	6.5	35.5–47.5
		Final	38	7	33–46
	Subpatellar	Initial	36.5	6	31–44
		Final	37.5	4.5	34–46
	Suprapatellar	Initial	44	6	40–56.5
		Final	46.5	6.5	43–58.5
Pain intensity		Initial	7	1	5–8
		Final	0	1	0–3
QoL	Mobility	Initial	3	0	2–4
		Final	1	0	1–2
	Self-care	Initial	1	1	1–3
		Final	1	0	1
	Usual activity	Initial	1	1	1–3
		Final	1	0	1
	Pain/discomfort	Initial	4	1	3–4
		Final	1	0	1–2
	Anxiety	Initial	1	0	1–2
		Final	1	0	1

**Table 3:** QoL dimensions before and after the rehabilitation program.

	Mobility		Self-Care		Usual Activities		Pain/Discomfort		Anxiety/Depression	
	Initial	Final	Initial	Final	Initial	Final	Initial	Final	Initial	Final
Level 1 (no problems)	0%	80%	57%	100%	66%	100%	0%	91%	69%	100%
Level 2 (slight problems)	17%	20%	34%	0%	23%	0%	0%	9%	31%	0%
Level 3 (moderate problems)	74%	0%	9%	0%	11%	0%	37%	0%	0%	0%
Level 4 (severe problems)	9%	0%	0%	0%	0%	0%	63%	0%	0%	0%
Level 5 (extreme problems/not able)	0%	0%	0%	0%	0%	0%	0%	0%	0%	0%

**Table 4:** Pearson Chi-square test for mobility.

	Chi-Square Tests for Mobility		
	Value	df	Asymptotic Significance (2-Sided)
Pearson Chi-Square	13.846 ^a^	2	0.001
Likelihood Ratio	12.703	2	0.002
Linear-by-Linear Association	9.000	1	0.003
N of Valid Cases	35		

^a^—Four cells (66.7%) have an expected count less than 5. The minimum expected count is 0.60.

**Table 5:** Pearson Chi-square test for pain.

	Chi-Square Tests for Pain/Discomfort				
	Value	df	Asymptotic Significance (2-Sided)	Exact Sig. (2-Sided)	Exact Sig. (1-Sided)
Pearson Chi-Square	1.939 ^a^	1	0.164		
Continuity Correction ^b^	0.589	1	0.443		
Likelihood Ratio	2.950	1	0.086		
Fisher’s Exact Test				0.279	0.235
Linear-by-Linear Association	1.884	1	0.170		
N of Valid Cases	35				

^a^—Two cells (50.0%) have an expected count less than 5. The minimum expected count is 1.11; ^b^—Computed only for a 2 × 2 table.

## Data Availability

The corresponding authors can provide with access to the data contained in this study upon request.

## References

[1] Astur D.C., Oliveira S.G., Badra R., Arliani G.G., Kaleka C.C., Jalikjian W., Golanó P., Cohen M. (2011). Updating of the Anatomy of the Extensor Mechanism of the Knee Using a Three-Dimensional Viewing Technique. Rev. Bras. Ortop..

[2] Manske R.C., Prohaska D. (2017). Rehabilitation Following Medial Patellofemoral Ligament Reconstruction for Patellar Instability. Int. J. Sports Phys. Ther..

[3] Hinckel B.B., Gobbi R.G., Kaleka C.C., Camanho G.L., Arendt E.A. (2018). Medial Patellotibial Ligament and Medial Patellomeniscal Ligament: Anatomy, Imaging, Biomechanics, and Clinical Review. Knee Surg. Sport. Traumatol. Arthrosc..

[4] Aframian A., Smith T.O., Tennent T.D., Cobb J.P., Hing C.B. (2017). Origin and Insertion of the Medial Patellofemoral Ligament: A Systematic Review of Anatomy. Knee Surg. Sport. Traumatol. Arthrosc..

[5] Masouros S.D., Bull A.M.J., Amis A.A. (2010). (I) Biomechanics of the Knee Joint. Orthop. Trauma.

[6] Krebs C., Tranovich M., Andrews K., Ebraheim N. (2018). The Medial Patellofemoral Ligament: Review of the Literature. J. Orthop..

[7] Koshino Y., Taniguchi S., Kobayashi T., Samukawa M., Inoue M. (2022). Protocols of Rehabilitation and Return to Sport, and Clinical Outcomes after Medial Patellofemoral Ligament Reconstruction with and without Tibial Tuberosity Osteotomy: A Systematic Review. Int. Orthop..

[8] Hayat Z., El Bitar Y., Case J.L. (2019). Patella Dislocation.

[9] Samelis P.V., Koulouvaris P., Savvidou O., Mavrogenis A., Samelis V.P., Papagelopoulos P.J. (2023). Patellar Dislocation: Workup and Decision-Making. Cureus.

[10] Guerrero P., Li X., Patel K., Brown M., Busconi B. (2009). Medial Patellofemoral Ligament Injury Patterns and Associated Pathology in Lateral Patella Dislocation: An MRI Study. BMC Sports Sci. Med. Rehabil..

[11] Dai R., Wu Y., Jiang Y., Huang H., Meng Q., Shi W., Ren S., Ao Y. (2024). Epidemiology of Lateral Patellar Dislocation Including Bone Bruise Incidence: Five Years of Data from a Trauma Center. Orthop. Surg..

[12] Manjunath A.K., Hurley E.T., Jazrawi L.M., Strauss E.J. (2021). Return to Play after Medial Patellofemoral Ligament Reconstruction: A Systematic Review. Am. J. Sports Med..

[13] Zaman S., White A., Shi W.J., Freedman K.B., Dodson C.C. (2018). Return-to-Play Guidelines after Medial Patellofemoral Ligament Surgery for Recurrent Patellar Instability: A Systematic Review. Am. J. Sports Med..

[14] Sporea C., Florescu M.S., Orzan O.A., Cristescu I. (2020). Improving the Perspectives on Quality of Life for Adolescents with Cerebral Palsy by Medical Textile. Ind. Textila.

[15] Kyung H.-S., Kim H.-J. (2015). Medial Patellofemoral Ligament Reconstruction: A Comprehensive Review. Knee Surg. Relat. Res..

[16] Dall’Oca C., Elena N., Lunardelli E., Ulgelmo M., Magnan B. (2020). MPFL Reconstruction: Indications and Results. Acta Bio. Medica Atenei Parm..

[17] Uimonen M.M., Repo J.P., Huttunen T.T., Nurmi H., Mattila V.M., Paloneva J. (2021). Surgery for Patellar Dislocation Has Evolved towards Anatomical Reconstructions with Assessment and Treatment of Anatomical Risk Factors. Knee Surg. Sport. Traumatol. Arthrosc..

[18] Flores G.W., de Oliveira D.F., Ramos A.P.S., Sanada L.S., Migliorini F., Maffulli N., Okubo R. (2023). Conservative Management Following Patellar Dislocation: A Level I Systematic Review. J. Orthop. Surg. Res..

[19] Halloran J.P., Esquivel A.O., Cracchiolo A.M., Chen C., Lemos S.E. (2020). The Role of the MPFL and MPTL in Patellar Stability—A Biomechanical Study. Arch. Orthop..

[20] Burcea C.-C., Bobu V., Ferechide D., Neagoe I.C., Lupușoru G.E., Sporea C., Denis Lupușoru M.O. (2023). New Methodological Aspects in Rehabilitation after Proximal Humerus Fracture. Balneo PRM Res. J..

[21] Sirbu E., Onofrei R.R., Hoinoiu T., Petroman R. (2021). The Short-Term Outcomes of Multiwave Locked System (MLS) Laser Therapy versus a Combination of Transcutaneous Nerve Stimulation and Ultrasound Treatment for Subacromial Pain Syndrome. Appl. Sci..

[22] Genah S., Cialdai F., Ciccone V., Sereni E., Morbidelli L., Monici M. (2021). Effect of NIR Laser Therapy by MLS-MiS Source on Fibroblast Activation by Inflammatory Cytokines in Relation to Wound Healing. Biomedicines.

[23] Diouf J.D., Diao S., Sy M.H., Gueye A.B., Kinkpe C.V.A., Niane M.M., Daffe M. (2018). Effects of Intermittent Dynamic Compression (Game Ready) on Treatment of Musculo-Skeletal Injuries: About 12 Basketball Professionals. J. Orthop. Rheumatol. Sport. Med..

[24] Klein I., Tidhar D., Kalichman L. (2020). Lymphatic Treatments after Orthopedic Surgery or Injury: A Systematic Review. J. Bodyw. Mov. Ther..

[25] Dehail P., Duclos C., Barat M. (2008). Electrical Stimulation and Muscle Strengthening. Proceedings of the Annales de Réadaptation et de Médecine Physique.

[26] Macedo L.B., Josué A.M., Maia P.H.B., Câmara A.E., Brasileiro J.S. (2015). Effect of Burst TENS and Conventional TENS Combined with Cryotherapy on Pressure Pain Threshold: Randomised, Controlled, Clinical Trial. Physiotherapy.

[27] Ribeiro S., Henriques B., Cardoso R. (2018). The Effectiveness of Tecar Therapy in Musculoskeletal Disorders. Int. J. Public Health Health Syst..

[28] Sorrentino M., Ferrari D., Elena Z.I. (2022). Effectiveness of a Long-Term Tecar Therapy Treatment on Knee Pain: Building TTESSK, an Evaluating Scale A Systematic Review and Meta-Analysis.

[29] Sbenghe T., Berteanu M., Săvulescu S.E. (2019). Kinetologie [Kinesiology].

[30] Firman G. (2009). Medical Research Council (MRC) Scale for Muscle Strength. https://www.criteria.blood.gov.au/NeurologicalScales/GeneratePDF?section=2.

[31] Crichton N. (2001). Visual Analogue Scale (VAS). J. Clin. Nurs..

[32] Hao K., Feng A., Kong L., Wang F. (2022). Quality of Life Following Medial Patellofemoral Ligament Reconstruction Combined with Medial Tibial Tubercle Transfer in Patients with Recurrent Patellar Dislocation: A Retrospective Comparative Study. J. Orthop. Surg. Res..

[33] EuroQol Group EQ-5D-5L. https://euroqol.org/information-and-support/euroqol-instruments/eq-5d-5l/.

[34] Massey F.J. (1951). The Kolmogorov-Smirnov Test for Goodness of Fit. J. Am. Stat. Assoc..

[35] Pallant J. (2020). SPSS Survival Manual: A Step by Step Guide to Data Analysis Using IBM SPSS.

[36] Oliveira A.G. (2020). Biostatistics Decoded.

[37] Lampros R.E., Wiater A.L., Tanaka M.J. (2022). Rehabilitation and Return to Sport after Medial Patellofemoral Complex Reconstruction. Arthrosc. Sport. Med. Rehabil..

[38] Fithian D.C., Powers C.M., Khan N. (2010). Rehabilitation of the Knee after Medial Patellofemoral Ligament Reconstruction. Clin. Sports Med..

[39] Skersick K. (2015). Rehabilitation Pre and Post MPFL Reconstruction Secondary to MPFL Tear.

[40] McNeilan R.J., Everhart J.S., Mescher P.K., Abouljoud M., Magnussen R.A., Flanigan D.C. (2018). Graft Choice in Isolated Medial Patellofemoral Ligament Reconstruction: A Systematic Review with Meta-Analysis of Rates of Recurrent Instability and Patient-Reported Outcomes for Autograft, Allograft, and Synthetic Options. Arthrosc. J. Arthrosc. Relat. Surg..

[41] Dai W., Leng X., Wang J., Cheng J., Hu X., Ao Y. (2022). Quadriceps Tendon Autograft versus Bone–Patellar Tendon–Bone and Hamstring Tendon Autografts for Anterior Cruciate Ligament Reconstruction: A Systematic Review and Meta-Analysis. Am. J. Sports Med..

[42] Ajrawat P., Dwyer T., Whelan D., Theodoropoulos J., Murnaghan L., Bhargava M., Ogilvie-Harris D., Chahal J. (2021). A Comparison of Quadriceps Tendon Autograft with Bone-Patellar Tendon-Bone Autograft and Hamstring Tendon Autograft for Primary Anterior Cruciate Ligament Reconstruction: A Systematic Review and Quantitative Synthesis. Clin. J. Sport Med..

[43] McGee T.G., Cosgarea A.J., McLaughlin K., Tanaka M., Johnson K. (2017). Rehabilitation after Medial Patellofemoral Ligament Reconstruction. Sports Med. Arthrosc..

[44] Morgan C., Bell R.M., Burland J.P., Kriscenski D., Ilinski A., Cote M.P., Edgar C.M. (2022). Effectiveness of an Accelerated Rehabilitation Protocol after Tibial Tubercle Osteotomy. Orthop. J. Sport. Med..

[45] Magnussen R.A., Peters N.J., Long J., Pappa N., Schmitt L.C., Brunst C.L., Kaeding C.C., Flanigan D.C. (2022). Accelerated Rehabilitation Program Following Medial Patellofemoral Ligament Reconstruction Does Not Increase Risk of Recurrent Instability. Knee.

[46] Hysing-Dahl T., Inderhaug E. (2024). Rehabilitation after Surgery for Patellar Instability. J. Exp. Orthop..

